# Protective Effects of Beta Glucan and Gliclazide on Brain Tissue and Sciatic Nerve of Diabetic Rats Induced by Streptozosin

**DOI:** 10.1155/2012/230342

**Published:** 2012-01-16

**Authors:** Harun Alp, Sefer Varol, Muhammet Murat Celik, Murat Altas, Osman Evliyaoglu, Orhan Tokgoz, Mehmet Halis Tanrıverdi, Ertugrul Uzar

**Affiliations:** ^1^Department of Pharmacology and Toxicology, Veterinary Faculty, Dicle University, 21103 Diyarbakir, Turkey; ^2^Department of Neurology, Faculty of Medicine, Dicle University, 21103 Diyarbakir, Turkey; ^3^Department of Internal Medicine, Faculty of Medicine, Mustafa Kemal University, 31000 Hatay, Turkey; ^4^Department of Neurosurgery, Faculty of Medicine, Mustafa Kemal University, 31000 Hatay, Turkey; ^5^Department of Biochemistry, Faculty of Medicine, Dicle University, 21103 Diyarbakir, Turkey; ^6^Department of Anesthesiology, Faculty of Medicine, Dicle University, 21103 Diyarbakir, Turkey; ^7^Department of Family Medicine, Faculty of Medicine, Dicle University, 21103 Diyarbakir, Turkey

## Abstract

There have not been yet enough studies about effects of beta glucan and gliclazide on oxidative stress created by streptozotocin in the brain and sciatic nerve of diabetic rats. The aim of this paper was to investigate the antioxidant effects of gliclazide and beta glucan on oxidative stress and lipid peroxidation created by streptozotosin in brain and sciatic nerve. Total of 42 rats were divided into 6 groups including control, diabetic untreated (DM) (only STZ, diabetic), STZ (DM) + beta glucan, STZ (DM) + gliclazide, only beta glucan treated (no diabetic), and only gliclazide treated (no diabetic). The brain and sciatic nerve tissue samples were analyzed for malondialdehyde (MDA), total oxidant status (TOS), total antioxidant status (TAS), oxidative stress index (OSI), and paraoxonase (PON-1) levels. We found a significant increase in MDA, TOS, and OSI along with a reduction in TAS level, catalase, and PON-1 activities in brain and sciatic nerve of streptozotocin-induced diabetic rats. Also, this study shows that in terms of these parameters both gliclazide and beta glucan have a neuroprotective effect on the brain and sciatic nerve of the streptozotocin-induced diabetic rat. Our conclusion was that gliclazide and beta glucan have antioxidant effects on the brain and sciatic nerve of the streptozotocin-induced diabetic rat.

## 1. Introduction

Diabetes mellitus (DM) is one of the most common chronic metabolic disorders leading to complications in a number of organ and systems. DM characterised by disturbed glucose metabolism due to an absolute or relative insulin deficiency affects the central and peripheral nervous systems [1]. It is reported that encephalopathy is the long-term neurological complication of diabetes and is associated with cognitive decline and increased risk of dementia [2]. Brain and nerve tissues are two sites of diabetic organ damage [3]. Also, it is reported that oxidative stress and apoptosis play an important role in diabetes-induced tissue damage [4, 5]. DM, in hyperglicemia uncontrolled, initiates degenerative processes that causes damage of brain and nerve tissues because of excess oxidative stress. In diabetic complications, oxidative stress results from an overproduction of reactive oxygen/nitrogen species generated by glucose autoxidation, mitochondria dysfunction, protein glycation and from decreased antioxidant defenses [6].

One of the important goals of DM treatment is to prevent its complications. To this end, there is accumulating evidence that supplementation antioxidant compounds may offer some protection against diabetic complications [7]. Therefore, beta-glucan and gliclazide were selected as possible protective materials of this study. Beta-glucans are glucose polymers that are found in the cell walls of yeast, fungi, and cereal plants. They are known to have beneficial effects on the immune system and are claimed to have no toxic or adverse effects. Natural products containing beta-glucans have been used for thousands of years for the benefits of human health, but beta-glucans were only identified as active components recently [8]. Beta-glucans have been investigated extensively for immune stimulation effects and developed for the treatment of several diseases including cancer, decubitus ulcer, and infectious diseases. Recent studies have reported that beta-glucans could reduce hyperglicemia, hyperlipidemia, and hypertension [9]. Several mechanisms were suggested for the protective effect of beta-glucan; one of these mechanisms is related to antioxidant capacity of this molecule. It was found that *β*-glucan is an antioxidant with the scavenging ability lying between that of *α*-tocopherol, which is known to be incorporated in the lipid bilayer, and the water-soluble antioxidant, mannitol [10]. Therefore, beta-glucans have great potential for the treatment of diabetes and associated neurological diseases including diabetic neuropathy and encephalopathy. Thus, beta glucan can lead new approaches for the prevention of diabetic neurologic complications and vascular risk factors by reducing oxidative damage of this molecule. However, there have not been yet enough studies for its antioxidant actions on diabetic brain and sciatic norve tissues. The aim of this study was to investigate the antioxidant protective effects of beta glucan on brain and sciatic nerve tissues of streptozotocin-induced diabetic rats.

Gliclazide which is another possible protective material of this study, is a second-generation sulfonylurea hypoglycemic agent. It may show effect as reduction in free radical generation or an increase in free radical scavenging. Also, gliclazide may contribute to the control of the physiopathological mechanisms underlying both the process of aging and type 2 diabetes by reducing oxidant stress and DNA damage, improving antioxidant status [11]. In diabetic experimental models, it has been reported that gliclazide potentially protects the vasculature through improvements in plasma lipids and platelet function [12]. However, there have not been yet enough studies about its antioxidant actions on diabetic brain and sciatic nerve tissues. Therefore, in the present study, the protective effects of gliclazide against oxidative stress and lipid peroxidation created by streptozotocin on brain and sciatic nerve in diabetic rats were investtigated.

## 2. Materials and Methods

### 2.1. Animals, Care, and Nutrition

This study was approved by Dicle University Animal Ethical Committee and was carried out in accordance with the ‘‘Animal Welfare Act and the Guide for the Care and Use of Laboratory animals prepared by the Dicle University, Animal Ethical Committee.” Female Wistar Albino rats (250 ± 50 g) were obtained from the Animal labaratuary of Dicle University. The rats were housed in clean polypropylene cages having six rats per cage and maintained under temperature controlled room (23 ± 2°C) with a photoperiod of 12 h light and 12 h dark cycle. The rats were given standard pellets diet and water ad libitum throughout the experimental period. All efforts were made to minimize animal suffering and to use only the number of animals necessary to produce reliable scientific data.

### 2.2. Animals and Treatment

The rats were fasted overnight, and diabetes was induced by a single dose via intraperitoneal injection of streptozotocin solution (STZ). The experiment was designed as a total of 28 days. A week after STZ administration, their blood glucose values were measured and were defined as diabetic rats, that is, their blood glucose values were above 250 mg/dL. STZ caused the death of some rats. At the end, 42 rats were divided into 6 groups including control, diabetic untreated (only STZ, diabetic), diabetics treated with beta glucan (STZ + beta glucan), diabetics treated with gliclazide (STZ + gliclazide), only beta glucan treated (beta glucan control, no diabetic), and only gliclazide treated (gliclazide control, no diabetic). The beta-glucan (Mustafa Nevzat Company, Turkey) used in this study is 1,3-1,6 beta-D-glucan in the microparticulate form, which was prepared from the S. cerevisiae yeast. Beta glucan was administered orally (50 mg/kg body weight) with a gavage for 21 days in the STZ + beta glucan and beta glucan groups. The gliclazide (DIAMICRON, Mustafa Nevzat Company, Turkey) used in the tablet form was administered orally (10 mg/kg body weight) with a gavage for 21 days in the STZ + gliclazide and only gliclazide groups. STZ solution was prepared as follows: 24 mg of STZ was dissolved in 1 mL of 5 mM citrate buffer (pH 4.5) before the injection, and a volume of 2.5 mL/kg was administrated into each rat. The animals were fasted overnight and diabetes was induced by a single i.p. injection of a freshly prepared solution of STZ (CAS number: 133 18883-66-4, 85882, Sigma-Aldrich) (50 mg/kg body weight) in 0.1 M cold citrate buffer (pH 4.5). One week after STZ administration, blood was taken from the lateral veins of the tail, their blood glucose levels were measured by a glucometer (ACCU-CHEK, Roche Diagnostics) using a glucose oxidase method and the rats whose blood glucose values were above 250 mg/dL were accepted as diabetic [13]. After completion of 21 days for drug treatments, the animals were sacrificed by cervical dislocation, and the brain and sciatic nerve tissues were excised at 4°C. The tissues were washed with ice-cold saline and immediately stored at −50°C for biochemical analyses.

### 2.3. Biochemical Analyses

The excised cerebrum and sciatic nerve tissue samples for biochemical analyses were weighed, immediately stored at −50°C. The cerebrum tissues cleaned with 1.15% ice-cold KCl, minced, then homogenized in five volumes (w/v) of the same solution. Assays were performed on the supernatant of the homogenate that is prepared at 14.000 rpm for 30 min at +4°C. The protein concentration of the tissue was measured by the method of Lowry [14]. Lipid peroxidation level in the cerebrum was expressed as malondialdehyde (MDA). It was measured according to procedure of Ohkawa et al. [15]. Catalase activity was measured according to the method of Aebi [16]. Serum paraoxonase (PON-1) activity was measured spectrophotometrically by modified Eckerson method [17]. The TAS of supernatant fractions was evaluated by using a novel automated and colorimetric measurement method developed by Erel [18]. Hydroxyl radicals, the most potent biological radicals, are produced in this method. In the assay, the ferrous ion solution present in reagent 1 is mixed with hydrogen peroxide, which is present in reagent 2. The subsequently produced radicals, such as brown-colored dianisidine radical cations produced by the hydroxyl radicals, are also potent radicals. Using this method, the antioxidative effect of the sample is measured against the potent-free radical reactions initiated by the produced hydroxyl radicals. The assay has excellent precision values lower than 3%. The total antioxidant status (TAS) results are expressed as nmol Trolox equivalent/mg protein. The total oxidant status (TOS) of supernatant fractions was evaluated by using a novel automated and colorimetric measurement method developed by Erel [19]. Oxidants present in the sample oxidize the ferrous ion-o-dianisidine complex to ferric ion. The oxidation reaction is increased by glycerol molecules, which are abundantly present in the reaction medium. The ferric ion makes a colored complex with xylenol orange in an acidic medium. The color intensity, which can be measured spectrophotometrically, is related to the total amount of oxidant molecules present in the sample. The assay is calibrated with hydrogen peroxide, and the results are expressed in terms of nmol H_2_O_2_ equivalent/mg protein [20]. The TOS level to TAS level ratio was regarded as the oxidative stress index (OSI). The unit of cerebrum tissue TOS and TAS was *μ*mole H_2_O_2_ Equiv./gram protein and mmole H_2_O_2_ Equiv./gram protein, respectively. The cerebrum tissue OSI value was calculated as follows: OSI = ((TOS, *μ*mole H_2_O_2_ Equiv./gram protein)/(TAS, *μ*mole H_2_O_2_ Equiv./gram protein) × 100) [21].

### 2.4. Statistical Analyses

The data was analyzed by using Statistical Package for the Social Sciences version 11.5 (SPSS 11.5 for Windows, Chicago, IL, USA). The variables between the groups were tested by Mann Whitney *U* test. A value of *P* < 0.05 indicates a significant difference. Data are expressed as mean ± S.D.

## 3. Results

In the rat brain, the MDA, TOS, TAS, and OSI level, catalase and PON-1 enzyme activities are presented in Table 1. There was a significant depletion in the PON-1, catalase, and TAS levels in the brain of the diabetic rat compared to the control groups (for both parameters *P* < 0.05). However, beta glucan-treated diabetic rats significantly reversed the catalase, PON-1 and TAS back to normal levels compared to untreated diabetic rats (for both parameters *P* < 0.05). PON-1 activity was significantly higher in gliclazide-treated diabetic group than untreated diabetic group in the brain (*P* < 0.05), but catalase activity and TAS level were not significant (for both parameters *P* > 0.05). As can be seen from Table 1, the level of MDA, TOS, and OSI in the brain tissue was increased in untreated diabetic rats compared with the rats of control group (for both parameters *P* < 0.05). However, MDA, TOS, and OSI levels were significantly reduced to gliclazide-treated diabetic group compared with the untreated-diabetic group in brain tissue (for both parameters *P* < 0.05). Likewise, MDA, TOS, and OSI levels were significantly reduced to beta-glucan-treated diabetic group compared with the untreated-diabetic group in brain tissue (for both parameters *P* < 0.05).

It was observed that medication similarly affected both tissue in the rat sciatic nerve compared with brain tissue. In the rat sciatic nerve, the MDA, TOS, TAS, and OSI levels as well as catalase and PON-1 enzyme activities are presented in Table 2. There were no significant differences in brain tissue MDA, TOS, TAS, OSI, catalase, and PON-1 in both beta glucan-treated group and gliclazide-treated group compared to control group rats (*P* > 0.05). There was a significant depletion in the PON-1, catalase, and TAS levels in the sciatic nerve of the diabetic rat compared to the control groups (for both parameters *P* < 0.05). However, beta-glucan-treated diabetic rats significantly reversed catalase, PON-1, and TAS back to normal levels compared to untreated diabetic rats (for both parameters *P* < 0.05). PON-1 activity was significantly higher in gliclazide-treated diabetic group than untreated diabetic group in the brain (*P* < 0.05), but catalase activity and TAS level were not significant (for both parameters *P* > 0.05). As can be seen from Table 2, the levels of MDA, TOS, and OSI in the sciatic nerve tissue were increased in untreated diabetic rats compared with the rats of control group (for both parameters *P* < 0.05). However, MDA, TOS, and OSI levels were significantly reduced to gliclazide-treated diabetic group compared with the untreated-diabetic group in sciatic nerve tissue (for both parameters *P* < 0.05). Likewise, MDA, TOS, and OSI levels were significantly reduced to beta-glucan-treated diabetic group compared with the untreated-diabetic group in sciatic nerve tissue (for both parameters *P* < 0.05).

## 4. Discussion

DM is a metabolic disorder with a globally rising prevalence which can affect the peripheral and central nervous system [2, 22]. It is well known that oxidative stress is a contributor to the development of complications in DM. There are several lines of evidence indicating that oxidative stress is increased in diabetic neuropathy and encephalopathy [23, 24]. Streptozotocin-induced diabetes is a well-described model of experimental diabetes that provides a relevant example of endogenous chronic oxidative stress as a result of hyperglycemia in diabetic brain [4].

Previous experimental studies in STZ-induced diabetic rats have been suggested that the increased oxidative stress is an important factor in the pathogenesis of the complications [4]. For example, increased lipid peroxidation impairs membrane function by decreasing membrane fluidity and causes free-radical-induced membrane lipid peroxidation including increased membrane rigidity, decreased cellular deformability, leading to various diseases [25]. Lipid peroxidation is initiated by free radicals attack to membrane lipids, generating large amounts of reactive products, which have been implicated in diabetes and its complications. MDA is a decomposition product of peroxidized polyunsaturated fatty acids, end product of lipid peroxidation. Thus, MDA is a marker of lipid peroxidation. In this study, the level of MDA significantly increased in the untreated diabetic rat sciatic and brain tissues. And it was concluded that the increase in lipid peroxidation might be a reflection of the decrease in enzymatic and nonenzymatic antioxidants of defense systems in diabetic rats. Similarly, in previous studies, increased MDA levels have been found in brain and sciatic nerve of diabetic rats [4, 26]. Also, in our study, the reduced MDA levels by both gliclazide and beta glucan likely demonstrate that beta glucan and gliclazide might be agents to protect the brain and nerve tissues against diabetic oxidative stress. In one study, it has been reported that treatment with gliclazide prevented the increase level of lipid peroxidation marker in plasma and pancreas of diabetic rats. In another a study, it has been found that MDA level decreased following gliclazide treatment in patients with diabetes [25, 27]. Also, it has been reported that gliclazide may be beneficial by inhibition of lipid and protein denaturation [28]. Similar to our study, Delibas et al. reported that gliclazide treatment prevented the elevation of MDA levels in hippocampus of diabetic rats [29]. In addition to MDA level, measurement of TOS, TAS, and OSI provides novel and reliable index of oxidative stress. The levels of oxidants can already be measured separately in the laboratory, but these measurements are time-consuming and costly. The number of different oxidants in all biological samples makes it difficult to measure each oxidant separately. Assessment of TOS may be indicating the level of all free oxidant radicals caused by diabetes-related oxidative stress [30]. Likewise, the number of different antioxidants in all biological samples makes it difficult to measure each antioxidant separately. Thereby, TAS may be an important factor providing protection from neurological damage caused by diabetes-related oxidative stress [31]. Therefore, we investigated both TAS and TOS by using new measurement methods developed by Erel (2004, 2005) to more accurately assess oxidative stress in this study [18, 19]. Also we evaluated oxidative stress with OSI, detected by using both TOS and TAS parameters. We found increased TOS and OSI levels and decreased TAS level and catalase activity in the diabetic rats compared to control rats in the brain and sciatic nerve tissues. At the same time, this study confirms that brain and sciatic nerve of diabetic rats show increased MDA, TOS, and OSI levels along with reduced TAS and catalase. These increases may be due to overproduction or decreased excretion of oxidant substances. Because of the increase in these oxidants and the decrease in total antioxidants, the oxidative/antioxidative balance demonstrated to shift towards the oxidative status in brain and sciatic nerve tissues of diabetic rats in this study. Treatment with beta glucan of diabetic rats was significantly reduced to TOS and OSI along with increased TAS level and catalase activity compared to untreated diabetic rats in brain and sciatic tissues. Because of the decrease in these oxidants and the increase in these antioxidants, the oxidative/antioxidative balance demonstrated to shift towards the antioxidative status with beta glucan treatment in brain and sciatic nerve tissues of diabetic rats. Additionally, there was no difference in beta glucan group between control group regarding sciatic and brain oxidant/antioxidant parameters. These findings show no toxic effect treatment with beta glucan in brain and sciatic nerve tissues of diabetic rats. Thus, it may be preferred to use natural products in treating and preventing various diseases.

Owing to their useful effects on the immune system, antioxidant, and anti-inflammatory properties, and because they lack any toxic effects, beta glucans have been used in previous many studies [32, 33]. It has been suggested that beta-glucans lowered postprandial glycemia, hyperlipidemia, and hypertension [34, 35]. Also, it is reported that beta glucan improves wound healing in diabetic mice [36]. In addition, it has been suggested that beta-glucans may be used to prevent or treat excessive microglial activation during chronic inflammatory conditions [37]. The present study is the first experimental research demonstrating the protective effectiveness of beta glucan to protect diabetes-induced oxidative stress in brain and sciatic nerve of rats.

Gliclazide which is a another marker of drug of the present study, second-generation sulfonylurea, might exert antioxidant effect. It has been demonstrated that gliclazide prevents, through its antioxidant properties, oxidized low-density lipoprotein-associated endothelial dysfunction, apoptosis, and plaque rupture [38]. It has been found that gliclazide was able to significantly reduce high glucose-induced apoptosis, mitochondrial alterations, and nitrotyrosine concentration increase [39]. It has been suggested that it decreases hyperglycemia and hyperinsulinemia and inhibits oxidative stress. It is also a promising therapeutic candidate for the prevention of vascular complications of diabetes, by decreasing the level of DNA damage induced by reactive oxygen species [13, 40]. Also it has been found that gliclazide reduced oxidative stress in liver and kidney tissues of diabetic rats [11, 41]. But the protective effect of gliclazide on diabetic brain and sciatic nerve tissues has not been reported so far, it is interested in whether antioxidative properties of gliclazide may occur to reduction in oxidative damage, in the context of prevention of diabetes-associated neuropathy and encephalopathy. In the present study, treatment with gliclazide of diabetic rats significantly reduced TOS and OSI levels compared to untreated diabetic rats in brain and sciatic tissues but insignificant increased TAS level and catalase activity. Because of the decrease in these oxidants and the increase in these antioxidants, the oxidative/antioxidative balance demonstrated to shift towards the antioxidative status with gliclazide treatment in brain and sciatic nerve tissues of diabetic rats. This study is the first experimental research demonstrating the effectiveness of gliclazide to protect diabetes-induced oxidative stress in brain and sciatic nerve of rats. As a result, we may explain these results due to the antioxidant and antidiabetic effects of gliclazide.

Paraoxonase is one of the important antioxidant enzymes. PON-1 hydrolyses lipid peroxides in oxidized lipoproteins. A close relationship between PON-1 deficiency and accelerated progression of arteriosclerosis has been found in experimental and human studies [42, 43]. Although microangiopathy plays a role in the pathogenesis of diabetic neuropathy, there has been little research on PON-1 activity in patients with DM. Therefore, PON-1 was selected as a marker agent of antioxidant defences system in present study. In a previous study, it has been found that serum PON-1 decreased in diabetic rats and spermine reversed the decline of PON-1 spermine-treated diabetic rats [44]. Also, it has been indicated that cerebrospinal fluid (CSF) levels of *β*-glucan and gliclazide correlated in a dose-dependent pattern with therapeutic response, and they penetrated to blood-brain barrier [45, 46]. To our knowledge, brain and sciatic nerve injury relationship between PON-1 activities has not been studied in diabetic rats. In this study, we found decreased PON-1 activity in the brain and sciatic tissues of diabetic rats compared to control rats. Both beta glucan and gliclazide treatments were reversed to decrement activity of PON-1 in diabetic rats.

## 5. Conclusions

Hyperglicemia in STZ-induced diabetic rats can cause oxidative damage in brain and sciatic nerve tissues, which may play vital roles in the pathogenesis of diabetic neuropathy and encephalopathy. Also treatment of beta glucan and/or gliclazide inhibits lipid peroxidation and regulates total oxidant/antioxidant status in diabetic rat brain and sciatic nerve tissues. This study results suggested that beta glucan and gliclazide may be considered to reduce oxidative stress in diabetic brain and sciatic nerve and may be used as a protective agent against diabetic damage of brain and sciatic nerve.

## Figures and Tables

**Table 1:** Biochemical parameters in all groups in the brain of rats.

Groups	MDA (nmol/gr protein)	TOS (mmol H_2_O_2_ Eq./g protein)	TAS (mmol Trolox Eq./g protein)	OSI	Catalase (U/g protein)	PON-1 activity (U/mg protein)
Control (I)	259.9 ± 60.5	22.0 ± 8.2	0.44 ± 0.1	53.6 ± 25.5	1.19 ± 0.14	0.72 ± 0.29
Diabetic (II)	454.7 ± 62.8	39.7 ± 3.5	0.22 ± 0.08	204.9 ± 96.3	0.65 ± 0.15	0.30 ± 0.05
Diabetic + gliclazide (III)	367.4 ± 47.6	32.2 ± 2.8	0.29 ± 0.04	113.7 ± 17.1	0.73 ± 0.12	0.40 ± 0.10
Diabetic + Beta glucan (IV)	329.2 ± 45.3	27.0 ± 5.9	0.38 ± 0.08	75.0 ± 26.9	1.02 ± 0.09	0.59 ± 0.26
Gliclazide (V)	242.0 ± 36.7	20.5 ± 6.3	0.44 ± 0.1	51.01 ± 28.7	1.34 ± 0.23	0.80 ± 0.18
Beta glucan (VI)	257.0 ± 55.0	21.8 ± 6.8	0.46 ± 0.11	52.7 ± 33.4	1.30 ± 0.26	0.71 ± 0.20
*P values*						
I-II	0.002	0.004	0.003	0.002	0.002	0.004
II-III	0.009	0.009	N.S.	0.009	N.S.	0.046
II–IV	0.002	0.004	0.012	0.002	0.002	0.016
I–VI	N.S.	N.S.	N.S.	N.S.	N.S.	N.S.
I–V	N.S.	N.S.	N.S.	N.S.	N.S.	N.S.

N.S: not significant, MDA: malondialdehyde, TOS: total oxidant status, TAS: total antioxidant status, OSI: oxidative stress index, PON-1: paraoxonase.

**Table 2:** Biochemical parameters in all groups in the sciatic nerve of rats.

Groups	MDA (nmol/gr protein)	TOS (mmol H_2_O_2_ Eq./g protein)	TAS (mmol Trolox Eq./g protein)	OSI	Catalase (U/g protein)	PON-1 activity (U/mg protein)
Control (I)	17.6 ± 3.1	11.4 ± 3.5	0.16 ± 0.01	70.7 ± 23.0	0.36 ± 0.07	0.11 ± 0.02
Diabetic (II)	30.2 ± 4.2	20.6 ± 1.9	0.08 ± 0.03	299.4 ± 161.9	0.19 ± 0.05	0.04 ± 0.01
Diabetic + gliclazide (III)	25.0 ± 2.9	17.7 ± 2.5	0.11 ± 0.02	162.3 ± 37.2	0.21 ± 0.03	0.06 ± 0.01
Diabetic + Beta glucan (IV)	23.0 ± 1.3	14.1 ± 2.7	0.14 ± 0.03	108.4 ± 41.5	0.29 ± 0.03	0.09 ± 0.03
Gliclazide (V)	16.1 ± 2.4	10.7 ± 3.2	0.16 ± 0.04	71.5 ± 40.8	0.39 ± 0.07	0.12 ± 0.03
Beta glucan (VI)	17.1 ± 3.6	11.3 ± 3.5	0.17 ± 0.04	73.6 ± 45.2	0.38 ± 0.08	0.10 ± 0.03
*P values*						
I-II	0.002	0.004	0.002	0.002	0.002	0.002
II-III	0.017	0.025	N.S.	0.018	N.S.	0.021
II–IV	0.002	0.004	0.012	0.002	0.002	0.006
I–V	N.S.	N.S.	N.S.	N.S.	N.S.	N.S.
I–VI	N.S.	N.S.	N.S.	N.S.	N.S.	N.S.

N.S: not significant, MDA: malondialdehyde, TOS: total oxidant status, TAS: total antioxidant status, OSI: oxidative stress index, PON-1: paraoxonase.
